# Impact of Anti-Angiogenic Treatment on Bone Vascularization in a Murine Model of Breast Cancer Bone Metastasis Using Synchrotron Radiation Micro-CT

**DOI:** 10.3390/cancers14143443

**Published:** 2022-07-15

**Authors:** Hao Xu, Marie-Hélène Lafage-Proust, Lamia Bouazza, Sandra Geraci, Philippe Clezardin, Bernard Roche, Françoise Peyrin, Max Langer

**Affiliations:** 1 Institute of Innovation Science and Technology, Shenyang University, Shenyang 110000, China; 2 CREATIS, Université de Lyon, CNRS UMR5220, INSERM, U1206, Université Lyon 1, INSA-Lyon, 69100 Villeurbanne, France; peyrin@esrf.fr (F.P.); max.langer@creatis.insa-lyon.fr (M.L.); 3 SAINBIOSE, Université de Lyon, INSERM, U1059, 42023 Saint-Etienne, France; mh.lafage.proust@univ-st-etienne.fr (M.-H.L.-P.); bernard.roche@univ-st-etienne.fr (B.R.); 4 INSERM, U1033, LYOS, Université de Lyon, 69000 Lyon, France; lamia.bouazza@univ-lyon1.fr (L.B.); sandra.geraci@univ-lyon1.fr (S.G.); philippe.clezardin@inserm.fr (P.C.); 5 European Synchrotron Radiation Facility (ESRF), 38000 Grenoble, France

**Keywords:** synchrotron radiation microcomputed tomography, breast cancer bone metastases, anti-angiogenic drugs, trabecular bone, vasculature

## Abstract

**Simple Summary:**

This study shows for the first time that the use of synchrotron radiation microcomputed tomography (SR-µCT) with a contrast agent enables the simultaneous quantitative measurement of three-dimensional (3D) bone and blood vessel microstructures in a murine model of breast cancer bone metastasis. Our data suggests that the combined anti-angiogenic drug (AAD) treatment (Bevacizumab + Vatalanib) dampens tumor-induced trabecular bone resorption and affects the 3D structure of bone microvascularization. The combined AAD might offer superior treatment to inhibit breast cancer bone metastasis in a mouse model.

**Abstract:**

Bone metastases are frequent complications of breast cancer, facilitating the development of anarchic vascularization and induce bone destruction. Therefore, anti-angiogenic drugs (AAD) have been tested as a therapeutic strategy for the treatment of breast cancer bone metastasis. However, the kinetics of skeletal vascularization in response to tumor invasion under AAD is still partially understood. Therefore, the aim of this study was to explore the effect of AAD on experimental bone metastasis by analyzing the three-dimensional (3D) bone vasculature during metastatic formation and progression. Seventy-three eight-week-old female mice were treated with AAD (bevacizumab, vatalanib, or a combination of both drugs) or the vehicle (placebo) one day after injection with breast cancer cells. Mice were sacrificed eight or 22 days after tumor cell inoculation (time points T1 and T2, respectively). Synchrotron radiation microcomputed tomography (SR-μCT) was used to image bone and blood vessels with a contrast agent. Hence, 3D-bone and vascular networks were simultaneously visualized and quantitatively analyzed. At T1, the trabecular bone volume fraction was significantly increased (*p* < 0.05) in the combined AAD-treatment group, compared to the placebo- and single AAD-treatment groups. At T2, only the bone vasculature was reduced in the combined AAD-treatment group (*p* < 0.05), as judged by measurement of the blood vessel thickness. Our data suggest that, at the early stage, combined AAD treatment dampens tumor-induced bone resorption with no detectable effects on bone vessel organization while, at a later stage, it affects the structure of bone microvascularization.

## 1. Introduction

Breast cancer is one of the most frequently diagnosed cancers in women worldwide [1]. In advanced disease, breast cancer cells can spread outside the primary tumor via the bloodstream and metastasize to distant organs, including bone, where they induce osteolytic lesions in most cases. Breast cancer bone metastases are associated with a poor prognosis, with survival rates for patients ranging from two to five years after bone involvement is diagnosed [2,3].

Angiogenesis is the formation of new blood vessels from preexisting vessels. In the 1970s, Judah Folkman introduced the concept of targeting tumor angiogenesis, in an effort to halt tumor growth [4]. After decades of preclinical and clinical trials, anti-angiogenic agents that target vascular endothelial growth factor (VEGF) or its receptors have been considered as a major anticancer treatment modality [5]. However, the clinical efficacy of anti-angiogenic therapies was more modest than originally thought, especially for breast cancer, and it required selection of patients to achieve a better clinical benefit [6,7,8]. Interestingly, there was experimental evidence that the combination of two anti-angiogenic molecules targeting VEGF (Bevacizumab) and its receptor (Vatalanib) reduced skeletal lesions in a mouse model of breast cancer bone metastasis, while either treatment alone had no inhibitory effect [9]. However, the mechanisms of skeletal vascularization in response to tumor invasion and the effects of anti-angiogenic drugs on bone metastasis formation are still poorly understood at the three-dimensional (3D) organ level. Thus, the blood vessel network topography and the kinetics of vessel development within bone metastases need to be more thoroughly examined.

So far, histology has been mainly used to assess angiogenesis in animal models of bone metastasis following immunostaining of blood vessels [10]. However, tumor-associated blood vessels in bone may be immature and lack proteins expressed by normal capillaries, and immunohistochemistry may not be sensitive enough to detect tiny blood vessels especially in the early steps of metastasis progression. In addition, 2D assessment of the microvasculature can only provide planar information and lacks 3D architectural information for assessing structural aspects of pathologies. Bioluminescent probes can provide global tracking of vascular invasion, but with poor spatial resolution [11]. Some researchers have used laser confocal scanning microscopy (LCSM) to image mouse bone marrow vasculature with immunofluorescence staining after bone decalcification of experimental breast cancer bone metastases [12]. Yet, it cannot provide information on volumetric tumor growth such as 3D spatial organization of the blood vessel network within the whole bone. Furthermore, LCSM can be limited by the field of view and depth of penetration, which is low in hard tissues such as bone.

3D X-ray micro-computed tomography (µCT) is a powerful tool to image bone vasculature with a contrast agent [13,14]. However, since the contrast between bone and blood vessels is weak, it generally requires decalcifying the bone prior to imaging. Thus, 3D X-ray µCT does not permit a simultaneous visualization of bone and blood vessels, thereby explaining why the relationships between blood vessels and bone proper remain poorly understood [15]. Synchrotron radiation µCT (SR-µCT) is an alternative tool to simultaneously visualize 3D bone and blood vessels infused with a contrast agent. This technique possesses the advantage of yielding a high contrast associated to a high spatial resolution and signal-to-noise ratio, compared to standard µCT [16]. SR-µCT has been previously proposed as a useful tool to analyze angiogenesis in long bones of rats [17,18] and mice [19,20]. It allows the detection of microvessels as small as 3µm in diameter and shows morphological changes in the bone microvascular network under physiological and pathological conditions. However, SR-µCT has so far never been applied to analyze 3D bone microvascular network in the context of bone metastases.

Following tissue imaging, image segmentation is an important step in view of performing quantitative analysis and a standard tool in biomedical research [21,22]. To be able to analyze the characteristics of blood vessels, the segmentation of the vasculature structure is a prerequisite. However, this step may be challenging due to the complexity of the vascular network and the lack of diffusion of the contrast agent at some locations. In addition, blood vessels may appear to be in close proximity to the bone surface in SR-µCT images, preventing the correct segmentation of bone and blood vessels. Recently, an improved region growing-based method was proposed to solve this issue [23].

3D imaging and segmentation open possibilities in the quantification of bone and blood vessels in preclinical models. In particular, quantitative parameters of bone volume fraction, bone thickness, and structure model index (SMI) have been widely used to evaluate the variation of bone in the context of breast cancer bone metastasis [24,25,26,27]. Furthermore, connectivity density (Conn.D) and Fractal dimension (FD) of trabecular bone have also been used to describe bone in 3D images [28,29,30].

With regard to quantitative parameters for blood vessels, volume fraction and thickness may be measured from SR-µCT images [17,18]. Previous studies used FD to analyze blood vessels in tumor angiogenesis and cerebral arteriovenous malformations using 2D images obtained by scanning electron microscopy [31] and magnetic resonance imaging (MRI) [32], respectively. Similarly, the number and morphology of blood vessels within bone metastases are often measured using 2D microscopy images [12,33,34,35]. In sharp contrast, 3D quantitative analysis of blood vessels has never been performed in any tissue, including bone.

In this context, the aim of this study was to analyze and quantify the morphological and functional traits of the bone microvascular network in a murine model of breast cancer bone metastasis. Specifically, we examined the impact of three different anti-angiogenic treatment regimens (Bevacizumab, Vatalanib, and Bevacizumab + Vatalanib) on bone metastasis formation and progression, compared with placebo. SR-μCT coupled with a contrast agent was used to simultaneously image bone and blood vessels in mouse tibiae. Image segmentation was then performed using the previously proposed marker-controlled watershed in conjunction with the monogenic signal phase asymmetry [23]. To quantify the bone microstructure and skeletal vasculature, we computed a few parameters, such as volume fraction, thickness, fractal dimension, structure model index, and connectivity density. Finally, the spatial relationship between blood vessels and trabecular bone in skeletal metastases has been investigated.

## 2. Methods

### 2.1. Sample Preparation

**Figure 1:**
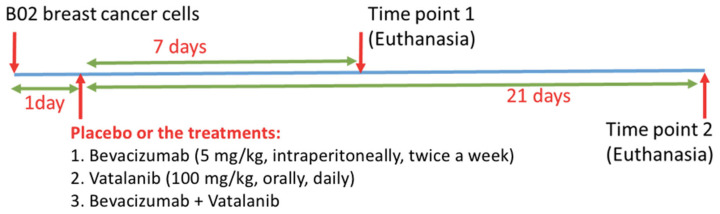
Summary of the treatment protocols for anti-angiogenic drugs in the mouse model of human B02 breast cancer metastasis to bone. One day after tumor cell inoculation, eight-week-old female Balb/c nude mice were treated with bevacizumab, vatalanib, a combination of both drugs, or the placebo. Animals were then sacrificed either 8 or 22 days after tumor cell inoculation.

Seventy-three eight-week-old female Balb/c nude mice underwent an intravenous injection of luciferase-expressing human B02 breast cancer cells [9]. One day after tumor cell inoculation, the placebo (P) or the treatments with different anti-angiogenic drugs (AAD) were given: Bevacizumab (B, 5 mg/kg, intraperitoneally, twice a week), Vatalanib (V, 100 mg/kg, orally, daily) and a combination of both drugs (C). Mice were sacrificed 8 or 22 days (namely T1 and T2, respectively) after tumor cell injection, as shown in Table 1. T1P, T1B, T1V and T1C groups stand for placebo, Bevacizumab, Vatalanib, and the combined treatment (Bevacizumab + Vatalanib) at T1, respectively. T2P, T2B, T2V, and T2C groups stand for placebo, Bevacizumab, Vatalanib, and the combined treatment at T2, respectively. The treatment protocols are shown in Figure 1. For vascular imaging, euthanized mice were infused through the left ventricle with a barium sulfate suspension (Micropaque, Guerbet, Paris, France), as previously reported [19]. The protocol (DR2015-18) was examined by the ethics committee (CNREEA C2EA-55) and approved by the Minister of Higher Education, Research and Innovation (Ministère français de 1’Enseignement supérieur, de la Recherche et de 1’Innovation, approval number: 2015121515281004).

### 2.2. Synchrotron Radiation Microcomputed Tomography (SR-µCT)

Following the sample preparation, the dissected mouse tibiae were dehydrated, fixed, and embedded in methyl methacrylate and imaged using SR-µCT. This imaging experiment was performed at the European Synchrotron Radiation Facility (ESRF), Grenoble, France, on the beamline ID19. A 2048 × 2048 pixels CCD-based detector with effective pixel size of 3.5 µm was used to record images at evenly spaced angles of view over a 360° rotation. The exposure time and X-ray energy were set to 0.15 s and 26 keV, respectively. The acquisition of 2000 radiographs of one sample lasted approximately 8 min. After the image acquisition, 3D images of 2000 × 2000 × 1200 voxels were reconstructed using a filtered back projection algorithm yielding a cylindrical field of view (FOV) of 7-mm diameter. The reconstruction was carried out using the ESRF in-house developed software PyHST [36]. The processing involved corrections that take into account beam inhomogeneity, response of the detector, and decrease of current during acquisition.

### 2.3. Image Pre-Processing

To keep the same orientation between the reconstructed bone structures, we manually re-orientated the 3D objects using the open source ITK-SNAP software. In particular, we opened two arbitrary bone samples in ITK-SNAP and used the Registration tool. Two bone objects can be rotated in Euler angles by manually spinning a wheel showing in the software window. Here, the purpose of rotation was to keep the bone long axis being parallel to the vertical axis, regardless of their initial orientations. Additionally, the position of the bone sample may need to be translated by simply dragging. To let the samples have a similar bone length, we cropped a new volume for each sample, and a bounding box was used on each volume for saving storage memory and speeding up computation. An original image and its pre-processing result were presented in Figure S1 (Supplementary-1). Here, we display 2D slices from the 3D volumes. We repeated the pre-processing procedure on all samples.

### 2.4. Image Segmentation

Bone and blood vessels in SR-μCT images were segmented using a monogenic signal phase based watershed algorithm [23]. In particular, the marker-controlled watershed was used to separate blood vessels and bone without the need for post-segmentation merging of regions. To initialize the watershed algorithm, markers of bone, blood vessels, and background were generated to achieve coverage of all connected components in each class and no false positives. In addition, for the generation of control surface, we proposed the use of the monogenic signal phase asymmetry to improve the edge detections at the bone and blood vessel interfaces, in which contrast was relatively weak compared to the bone-background and the vessel-background interfaces.

### 2.5. Enhancement of the Vascular Network

After segmenting blood vessels, we observed apparent leaks from ruptured vasculature in some images as illustrated by the red arrow in Figure S2a (Supplementary-1). The problem might be caused by inadequate or inappropriate filling of contrast agent during the perfusion procedure.

A 3D line enhancement filter named “Tubeness”, developed to enhance line structures, was used to remove features other than lines, such as leaks of contrast agent [37]. Specifically, the filter is based on the eigenvalues of the Hessian matrix, the elements of which are obtained by convolutions with the second derivatives of a gaussian. The standard deviation of the gaussian is used to tune the filter response to specific line widths. In this study, a multi-scale integration of the filter responses to various widths was computed by adding the different single-scale filter responses. The resulting filtered images provide an improved segmentation and visualization of blood vessel structures, as shown in Figure S2b (Supplementary-1).

### 2.6. Quantitative Parameters

After 3D imaging and segmentation, quantitative parameters of bone and blood vessels, such as volume fraction, local thickness, fractal dimension (FD), structure model index (SMI), and connectivity density (Conn.D) were calculated.

#### 2.6.1. Volume Fraction

Volume fraction is an important parameter to characterize the relative volume of the different structures. In this study, we computed the following volume fractions, BV/TV, VV/TV, and Tb.BV/Mar.V, with the following notations:

- Bone volume (BV): the entire bone volume (cortex + trabeculae).
- Vessel volume (VV)
- Total volume (TV): volume inside the outer contour of the cortical bone.
- Trabecular bone volume (Tb.BV)
- Marrow cavity volume (Mar.V)

BV and VV were measured by counting voxels on the automatically segmented bone and blood vessels, respectively. In trabecular bone, it was difficult to visually estimate the contour and number of metastases in many cases, since trabeculae were fully eroded by the metastatic process, as Figure S3 (Supplementary-1).

TV was measured by generating the convex envelop of the cortical bone containing all voxels inside the outer contour of the cortical bone. Here, we used Avizo 9.7 (Thermo Scientific), which is a commercial software for 3D image visualization, analysis and modeling. We assumed that the changes from slice-to-slice were small, so we interpolated the marked regions between slices. Firstly, we manually marked the region inside the outer contour of the cortical bone using the 2D brush (painting) tool, at the first slice of a 3D volume. Then, the process was repeated every 50th slice until the whole volume was scanned. Next, the marked regions were interpolated linearly between slices. A transverse and sagittal slice of an original image and the generated total volume are illustrated in Figure S4 (Supplementary-1). Finally, the value of TV was measured by counting voxels on total volume.

The marrow cavity is the central cavity of bone. In this study, we separated the marrow cavity from bone by eroding the total volume until approaching the inner contour of the cortical bone. Then, trabecular bone was separated from cortical bone by masking the marrow cavity with the automatically segmented bone compartment. The value of Mar.V, Tb.BV were measured by counting voxels. The resulting marrow cavity, trabecular bone, and cortical bone on one slice are shown in Figure S5 (Supplementary-1).

#### 2.6.2. Local Thickness

The local thicknesses of the segmented compartments were calculated using the so-called sphere method [17,38]. The local thickness is defined at each point of a volume, as the diameter of the largest sphere centered on that point that fits inside the compartment. We used a homemade implementation of the method [39] to extract the local thickness of trabecular bone and blood vessels. Finally, we computed the mean trabecular thickness (Tb.Th) and mean vessel thickness (V.Th).

#### 2.6.3. Fractal Dimension

Fractal dimension (FD) is a mean of quantifying complex structures indicating that structures tend to fill space at different scales. The box-counting method is commonly used to compute fractal dimension, due to the easiness of its implementation [40].

The box-counting algorithm can be extended to 3D space domain to quantify the complexity of 3D objects. The surface of 3D structure is overlaid by different 3D cube meshes with varying cube sizes. The number of cubes containing the surface is calculated for each cube size. The 3D fractal dimension FD is then obtained from log(N(λ))=−FD·log(λ)+log(α) where N(λ) is the number of cubes, λ is the corresponding box size and α is a proportionality constant [40]. 3D box counting has been widely used in the analysis of trabecular bone [30,40] and 3D vasculature [41,42]. In this study, the 3D box counting algorithm was implemented to quantify 3D fractal complexity.

#### 2.6.4. Structure Model Index

The structure model index (SMI) was proposed to assess the plate-like or rod-like nature of trabecular bone. The SMI value, which is independent of the physical dimensions, is 0 for an ideal plate structure, and 3 for an ideal rod structure, and lies between 0 and 3 for a mixed structure of plates and rods. The SMI was originally defined as a function of volume V, surface area S and first derivative of the specific surface area $S^{\prime }$ as: $\mathrm{SMI}=6\cdot \frac{S^{\prime }\cdot V}{S^{2}}$. In the original method, the derivative is estimated and computed using a surface meshing [43]. An alternative computation of the SMI relying on the Steiner formula can be performed by using the following expression $\mathrm{SMI}=12\frac{V\cdot M}{S^{2}}$, where M represents integral of mean curvature [44]. In this work, we used this later method to evaluate the SMI.

#### 2.6.5. Connectivity Density

The connectivity is the maximum number of connections that can be removed without separating the structure into two connected sub-structures. It can be calculated from the Euler characteristic [45,46]. The connectivity density (Conn.D) is defined by dividing the connectivity by the total volume.

### 2.7. Statistical Analysis

To test the normality of the data in each group, the Lilliefors test was used. In this study, since only very few groups were non-normal, and on inspection they were not bimodal, we assumed all data come from a normally distributed population. To test homoscedasticity, we used Bartlett’s test. According to the test, although there were few groups coming from populations with unequal variances, we assumed all groups had the same variance. ANOVA was used to test whether there was a significant difference among treatment groups. The effect sizes after ANOVA test were calculated using Eta squared ($\eta^{2}$) [47]. The benchmarks were defined as small ($\eta^{2}=0.01$), medium ($\eta^{2}=0.06$), and large ($\eta^{2}=0.14$), according to Cohen’s guidelines [48]. In case a significant difference between groups was found, a post-hoc Tukey’s HSD test was applied to identify which group was significantly different from the others, following the ANOVA test. Additionally, we used Grubbs’s test to detect outliers in each group. P values less than 0.05 were considered statistically significant.

## 3. Results

### 3.1. 3D Displays of the Segmented Bone and Blood Vessels

**Figure 2:**
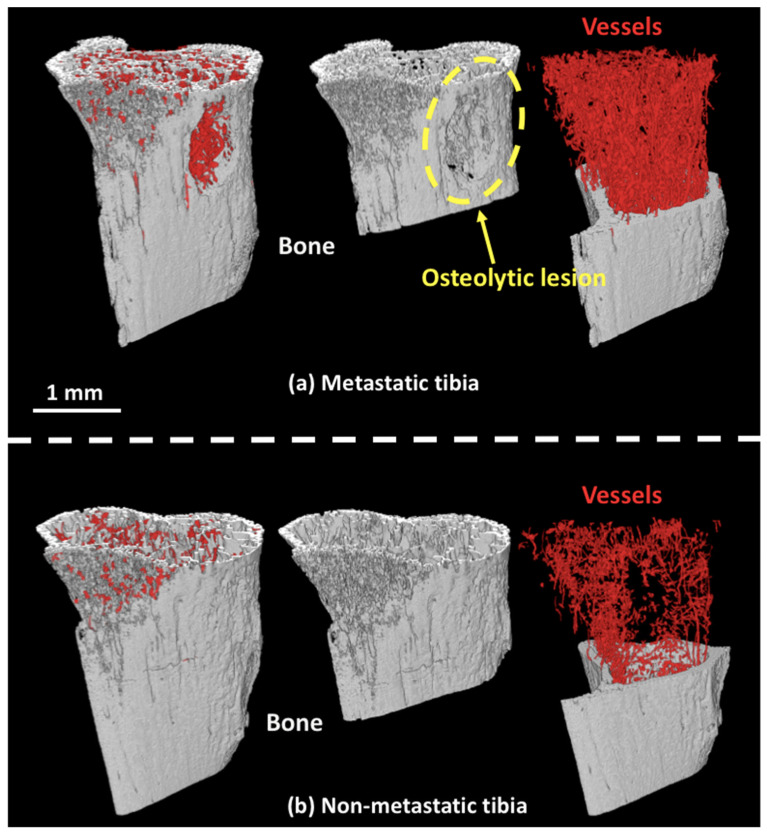
(**a**) 3D rendering of the segmented bone (gray) and blood vessels (red). (**b**) Same as in (**a**) for non-metastatic bones.

As exemplified in Figure 2a, the 3D rendering of the segmented bone and blood vessels of bone metastatic samples showed evidence of large osteolytic lesions that were highly vascularized as opposed to what was observed in non-metastatic bone samples (Figure 2b).

**Figure 3:**
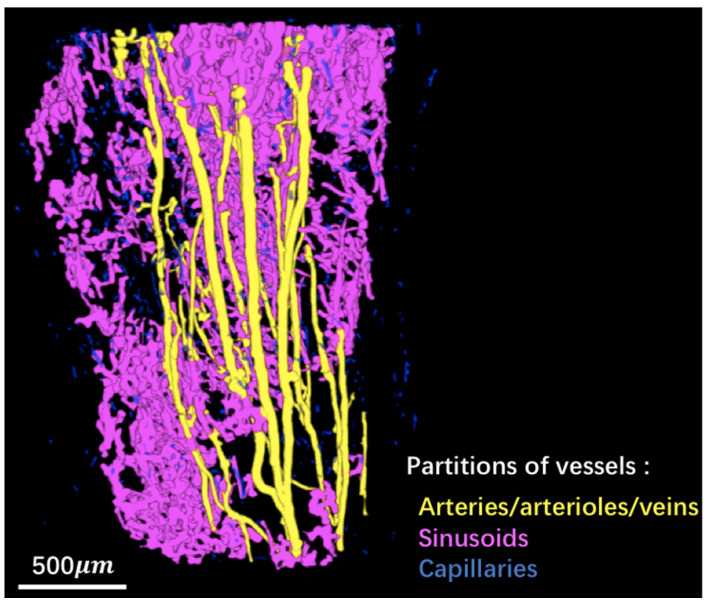
Partitions of blood vessels: arteries/arterioles/veins (yellow), sinusoids (magenta), and capillaries (blue).

In order to get some insights regarding the structure of the vascular network, we tried to partition it in different components: arteries/arterioles/veins, sinusoids and capillaries. To this aim, we applied image segmentation tools based on the local thickness map and connected component analysis. Figure 3 illustrates the demarcation of various types of bone marrow vessels after the process. Blood vessels were separated into arteries/arterioles/veins (yellow), sinusoids (magenta), and capillaries (blue). This example shows the possibility of classifying different types of blood vessels within the whole vascular network. An animation can be found in the supplementary material (Supplementary-2).

### 3.2. 3D-Measurements of Entire Bone and Blood Vasculature

**Figure 4:**
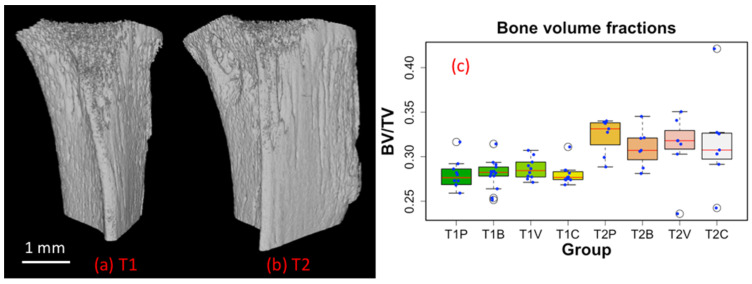
(**a**) 3D rendering of the segmented bone at the first time point (T1). (**b**) Same as in (**a**) at the second time point (T2). (**c**) Measurement of bone volume fraction (BV/TV). All data points (blue) were plotted along with the box plots. T1 and T2 denote time points at day 8 and 22 post tumor cell inoculation, respectively (P: Placebo, B: Bevacizumab, V: Vatalanib, C: Combination of Bevacizumab and Vatalanib).

We found that the BV/TV was significantly higher at T2 than at T1 irrespective of the treatment regimens (Figure 4). This bone gain is likely explained by the fact that the mice were young and in a rapid growth phase. When analyzing the entire bone (cortex + trabeculae), we did not find any difference in BV/TV between groups at either time point.

**Figure 5:**
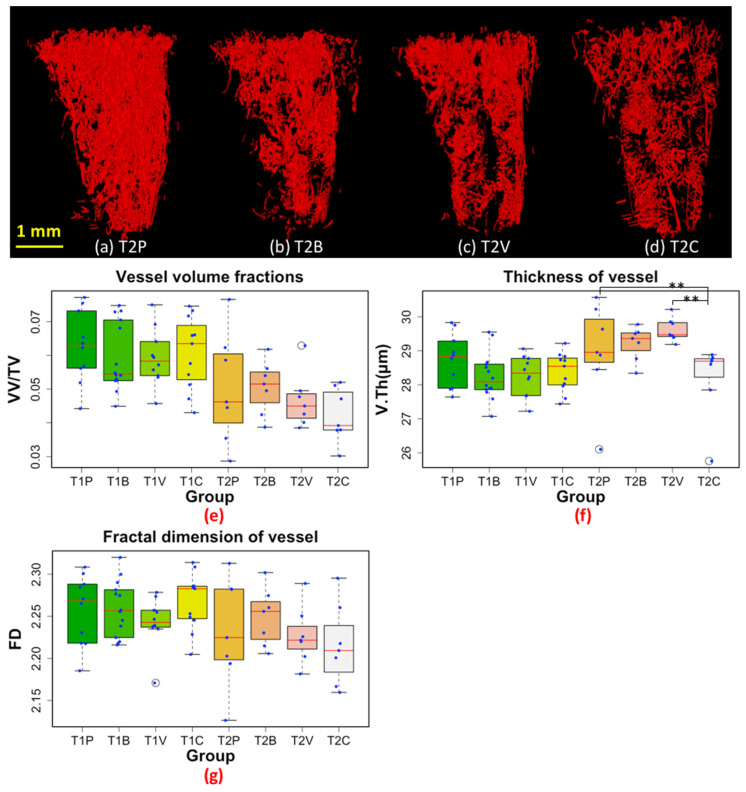
(**a**–**d**) 3D rendering of the segmented blood vessels at T2. (**e**) Boxplot of vessel volume fractions (VV/TV). (**f**) Boxplot of average vessel thickness (V.Th). (**g**) Boxplot of fractal dimension (FD) of blood vessels. Outliers are indicated with circles. The red line in each box represents the median value for the data set. Asterisk ** denotes statistically significant difference, at *p* = 0.05 level. All data points (blue) were plotted along with the box plots. T1 and T2 denote time points at day 8 and 22 post tumor cell inoculation, respectively (P: Placebo, B: Bevacizumab, V: Vatalanib, C: Combination of Bevacizumab and Vatalanib).

With regard to the skeletal vasculature, we did not find any inhibitory effect of the different anti-angiogenic therapies at T1, compared to placebo (Figure 5). This might be because T1 corresponds to a relatively early phase of bone metastasis development. At T2, we found that V.Th was significantly lower in the T2C group compared to T2P, with a non-significant trend to a decrease in VV/TV in T2C as well, while no such trend was observed in the T2B and T2V groups, suggesting that the full blocking of the VEGF signaling pathway is necessary to affect bone blood vasculature during the metastatic process, as suggested by our previous work [9].

### 3.3. 3D-Measurements of the Trabecular Bone in Tibial Metaphysis

**Figure 6:**
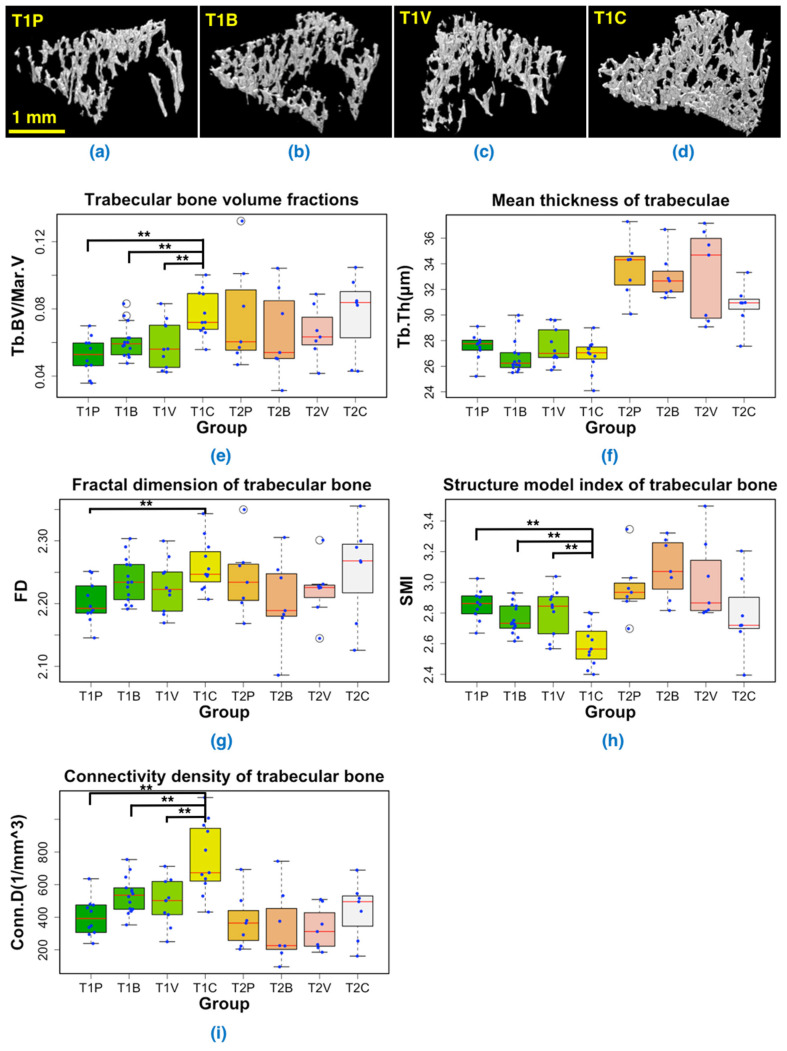
Analysis of trabecular bone in the tibial metaphysis. (**a**–**d**) 3D renderings of trabecular bone in T1P, T1B, T1V, and T1C. (**e**) Boxplot of trabecular bone volume fractions (Tb.BV/Mar.V). (**f**) Boxplot of the trabecular bone thickness (Tb.Th). (**g**) Boxplot of fractal dimension (FD). (**h**) Boxplot of structure model index (SMI). (**i**) Boxplot of connectivity density (Conn.D). Asterisk ** denotes statistically significant difference, at *p* = 0.05 level. Individual data are plotted as blue dots within the box plots. T1 and T2 denote the first and second time points, respectively. (P: Placebo, B: Bevacizumab, V: Vatalanib, C: Combination of Bevacizumab and Vatalanib).

Representative images of the 3D rendering of trabecular bone for the four different treatment groups at T1 are shown in Figure 6a–d. All 3D renderings of trabecular bone in the different groups at T1 and T2 are shown in Supplementary-3.

At T1, a combined AAD treatment substantially increased the trabecular bone volume fraction (*p* < 0.05) when compared to placebo or a single AAD treatment (Figure 6e). This increase in trabecular bone volume fraction was due to higher fractal dimension and/or connectivity density (*p* < 0.05), as other bone trabecular parameters (thickness, SMI) remained unchanged or were decreased, compared to placebo (Figure 6f–i). Here, significantly lower SMI value in the combined AAD treatment group indicated that there were more plate-like trabecular bone structures in the group, due to the reduced bone lesions by drug treatment (Figure 6h).

At T2, irrespective of the treatment groups, the trabecular bone volume fraction increased compared to that observed at T1 (Figure 6e). This increase of the trabecular bone volume fraction was associated with an increase in trabecular thickness (Figure 6f). It is highly likely that, compared to T1, the overall increase in trabecular bone volume fraction at T2 was related to the use of growing animals in our bone metastasis model. Moreover, under these experimental conditions, a combined AAD treatment did not any more increase the trabecular bone volume fraction (Figure 6e). It is possible however that the positive effect of a combined AAD treatment on trabecular bone was masked by the overall gain in trabecular bone associated with animal growth.

**Figure 7:**
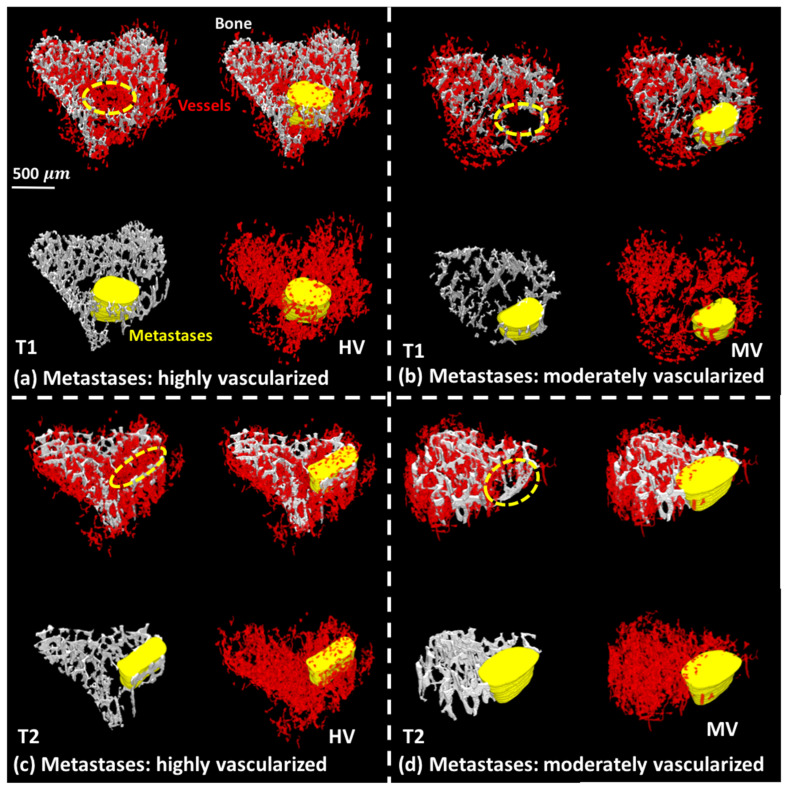
Illustration of spatial relationships between metastases (yellow) on trabecular bone (gray) and blood vessels (red). T1, metastases: (**a**) highly vascularized (HV), (**b**) moderately vascularized (MV); T2, metastases: (**c**) highly vascularized (HV), (**d**) moderately vascularized (MV).

Figure 7 shows 3D rendering to illustrate the spatial relationships between metastases, trabecular bone and blood vessels. In this study, trabecular bone (gray) was destroyed by metastases (yellow) as seen in the lower left part of Figure 7a–d. We observed two situations where in some cases metastases could be highly vascularized (HV) as in Figure 7a,c, or more modestly vascularized (MV) as in Figure 7b,d. All the 3D rendering of samples are given in Supplementary-4. This intrinsic heterogeneity of the vascularization of bone metastases from one specimen to another could account for the observed lack of efficacy of a single AAD treatment in animals.

## 4. Discussion

In the present study, we showed for the first time that the use of SR-µCT with a contrast agent enables the simultaneous quantitative measurement of 3D bone and vessel microstructures, providing an unbiased method to assess the efficacy of targeted therapies in a mouse model of bone metastasis.

The acquired 3D SR-µCT images were segmented into blood vessels, bone and background compartments using the marker-controlled watershed algorithm in conjunction with the monogenic signal phase asymmetry [23]. The image segmentation in our study was complex, not only due to the complexity of the vascular network, but also due to the lack of diffusion of the contrast agent at some locations. In addition, we addressed the problem of the structures not being spatially separate, which makes previous region-growing based protocol non-applicable. Specifically, the marker-based watershed was selected explicitly to find the intersections between the two structures, not to avoid merging of regions, since the structures can be relatively reliably separated by hysteresis thresholding to generate seeds. On the other hand, although the control surface for the watershed is often generated by the magnitude of the gradient, the contrasts between bone and blood vessels were relatively weak leading to weak edge detections at these boundaries. Therefore, we chose to use the phase asymmetry of monogenic signal, which is invariant to signal amplitude, to generate control surface.

We found that the BV/TV ratio, a measure of the bone volume, was significantly higher at T2 (22 days after tumor cell inoculation) than at T1 (eight days after tumor cell inoculation), irrespective of the treatment regimens (Figure 4). This bone gain was likely explained by the fact that mice were young growing animals. When analyzing the entire bone, we did not find any difference in the BV/TV ratio between the placebo and treatment groups at either time point. In contrast, when extracting data from the trabecular bone only (Figure 6), we observed that a combined AAD therapy preserved bone mass and structural bone parameters at T1, as opposed to what was observed with the placebo and AAD monotherapies. It has previously been reported that bone-resorbing osteoclasts express VEGF receptors VEGFR1 and R2, and that tumor-derived VEGF (in the presence of RANKL) stimulates osteoclast-mediated bone resorption in vitro [49]. It is therefore highly likely that a combined therapy targeting both VEGF (bevacizumab) and its receptors (vatalanib) prevented cancer-induced trabecular bone loss in our bone metastasis model by inhibiting osteoclast-mediated bone resorption. This beneficial effect was however lost at T2. Of note, using the same mouse model of bone metastasis and anti-angiogenic drugs, we have previously shown by histomorphometry that a four-week combined therapy with bevacizumab and vatelanib did not modify the BV/TV ratio, compared to placebo [9]. It is possible that the preventative effect on bone loss upon treatment with a combined anti-angiogenic therapy was masked by the animal growth-related gain in trabecular bone, thereby explaining the results obtained at T2.

With regard to the skeletal vasculature, we did not find any inhibitory effect of the different anti-angiogenic therapies at T1, compared to placebo (Figure 5e–g). This might be because T1 corresponds to a relatively early phase of bone metastasis development, so AAD administration did not target the appropriate signaling pathway, or the blood vessel sprouting from pre-existing vessels was not advanced enough. At T2, however, we found that a combined anti-angiogenic therapy substantially decreased the vessel thickness and, to a lower extent, the vessel volume, compared to a single-agent therapy or the placebo. This was in complete agreement with our previous study showing that a combined anti-angiogenic therapy targeting both VEGF and its receptors is superior to single-agent therapy to interfere with progression of bone metastases in an animal model of breast cancer [9]. In addition, when we combined bone and blood vessel 3D images, we observed that some metastases were highly irrigated while others, on the contrary, had a rather modest vascularization (Figure 7). This, which needs to be further investigated, suggests that the vascularization of bone metastases could be heterogeneous, as suggested by the recent discovery of vessel co-option. Vessel co-option is a non-angiogenic process whereby tumor cells directly utilize the pre-existing vasculature of the non-malignant tissue that they colonize [50], and this may partly explain the poor efficacy of an AAD monotherapy.

Vessel thickness is highly related to the types of blood vessels in the bone marrow [51]. Indeed, beside the relatively large nutrient arteries and drainage veins that are located at the center of the long bones, the normal bone marrow vascularization can be divided into large venous sinusoids organized in a mesh-like radial network, small arterioles which are straight and parallel to the bone axis and tiny transitional capillaries that are close to the bone surface. It is likely that developing bone metastases only affect venous sinusoids and arterial capillaries (which express different molecules and bear different niche cells on their wall) and do not alter central veins and arteries. Interestingly, these three types of blood vessels can be clearly observed in Figure 3 after image processing according to vessel size and shape. Thus, our data suggest that SR-µCT imaging of the microvessel network in the bone marrow could provide valuable information on dynamics and types of vessels and their response to AAD treatment during the metastatic process.

One of the advantages of our technique is that it allows to image the entire bone in a relatively short period of time, while microscopy-based imaging generally samples smaller ROIs. This might be of interest since it has been shown that some vessel-related events in the marrow microenvironment are discrete, with marrow sub regions dedicated to specific functions [52]. In addition, we could argue that looking at the whole bone marrow microenvironment (including the blood vessels) was shown to be as informative as what is observed within the metastases [53]. The SR-µCT has been validated with histology in our previous work [9], in which bone histology and histomorphometric analysis of bone tissue sections were performed using the same mouse model of bone metastasis and anti-angiogenic drugs as this study, supporting the reliability of the results of this study.

This study has several limitations. For many vascular parameters, we found trends between groups that did not reach statistical significance. Indeed, the effect sizes were large according to Cohen’s guidelines [48], and no significant differences were possibly due the small size of the groups. This may be the result of high inter-individual variability in terms of propensity to develop bone metastasis in this model, that could be prevented by increasing the number of animals per group. A second factor of variability is that, in this model, the tumor-induced angiogenesis may lead to the development of anarchic and non-mature neovessels characterized by increased leakiness of the contrast product outside of the vascular compartment. Finally, the mice were rather young (eight-week-old at the time of injection of breast cancer cells) compared to previous studies [19,20], leading to some difficulties to fully infuse the contrast agent into bone vascular network after heart catheterization [54]. We agree that this may also yield to missing blood vessels and a lack of continuity in the blood vessels on SR-µCT images. It is thus possible that the calculated vascular parameters did not show effects of AAD on angiogenesis at T1. However, this effect is not related to the SR-µCT imaging technique itself, which can offer even better spatial resolution than 3.5 µm, but is more related to the circulation of the contrast agent, thus making it difficult to quantify. Nevertheless, in previous studies, using the same perfusion protocols in rats permitted to assess physiological changes [18].

## 5. Conclusions

In conclusion, we show here that combining vessel contrasting and SR-µCT imaging with image processing allows us to quantitatively measure the 3D bone and vessel microstructures in a mouse model of bone metastasis and to analyze their spatial relationships. Our data suggest that, at the early stage, combined AAD treatment dampens tumor-induced bone resorption with no detectable effects on bone vessel organization while, at a later stage, it affects the structure of bone microvascularization and its relationship with the trabecular bone.

## Figures and Tables

**Table 1:** Experimental groups according to the time points and types of treatment.

	Treatments	Placebo	Bevacizumab	Vatalanib	Combination (Bevacizumab + Vatalanib)
Time Point (T) Number of Mice (n)					
T1 (day 8 after tumor cell injection) n		T1P 10	T1B 14	T1V 10	T1C 11
T2 (day 22 after tumor cell injection) n		T2P 7	T2B 7	T2V 7	T2C 7

## Data Availability

The data that support the findings of this study are available from the corresponding author upon reasonable request. Some data may not be made available because of privacy or ethical restrictions.

## Supplementary Materials

The following supporting information can be downloaded at: https://www.mdpi.com/article/10.3390/cancers14143443/s1, Supplementary-1, Figure S1: Illustration of image pre-processing. The left figure represents a sagittal slice in the original 3D image. The right one stands for the pre-processing result, after reorientation, cropping and bounding box. Figure S2: “Tubeness” enhancement of the 3D vascular network. (a) original image, in which sinusoids appear buried due to the leaks of contrast agent as indicated by red arrow; (b) vascular network with “Tubeness” enhancement, in which the leaks and the recovery of sinusoids are improved as indicated by red arrow. Figure S3: Illustration of metastases on trabecular bone. Figure S4: Slices of a 3D original image (left) and the generated total volume (right). Figure S5: Illustration of the resulting marrow cavity (red), trabecular bone (yellow), and cortical bone (blue) in one slice. Supplementary-2, video: Partitions of blood vessels: arteries/arterioles/veins. Supplementary-3: 3D renderings of trabecular bone in different groups at time point 1 and time point 2. Supplementary-4: 3D rendering of trabecular bone and blood vessels in different groups at time point 1 and time point 2.
