# Supplementary material for: Impact of Anti-Angiogenic Treatment on Bone Vascularization in a Murine Model of Breast Cancer Bone Metastasis Using Synchrotron Radiation Micro-CT
Source: Cancers (Basel). 2022 Jul 15;14(14):3443. doi: 10.3390/cancers14143443 (PMC9321934; doi:10.3390/cancers14143443)
Supplement: Supplementary file 1 [file cancers-14-03443-s001.zip › Supplementary/Supplementary-1.pdf]

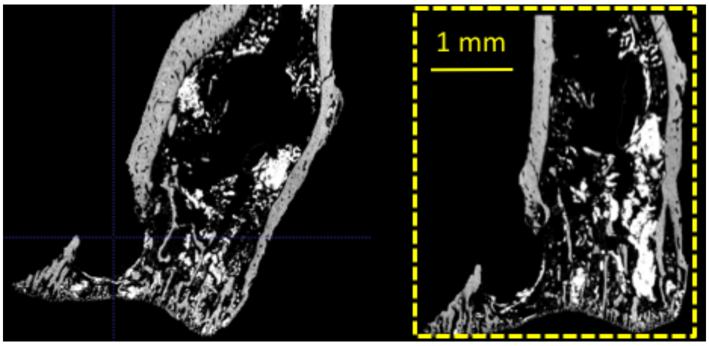

Fig. S1 Illustration of image pre-processing. The left figure represents a sagittal slice in the original 3D image. The right one stands for the pre-processing result, after reorientation, cropping and bounding box

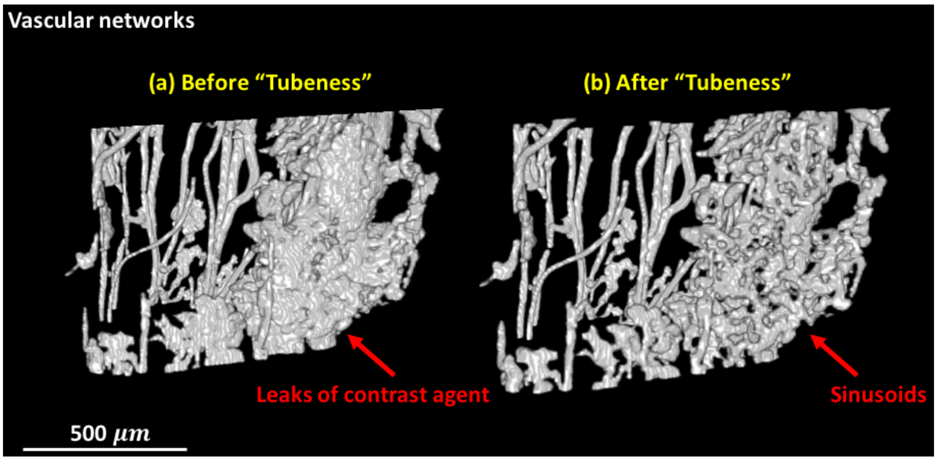

Fig. S2 "Tubeness" enhancement of the 3D vascular network. (a) original image, in which sinusoids appear buried due to the leaks of contrast agent as indicated by red arrow; (b) vascular network with "Tubeness" enhancement, in which the leaks and the recovery of sinusoids are improved as indicated by red arrow

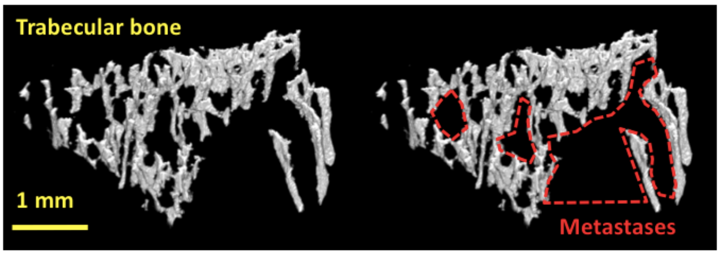

Fig. S3 Illustration of metastases on trabecular bone

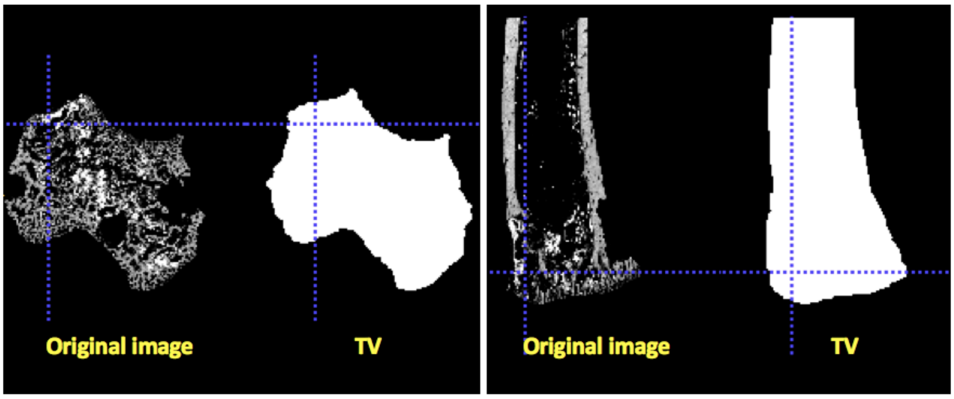

Fig. S4 Slices of a 3D original image (left) and the generated total volume (right)

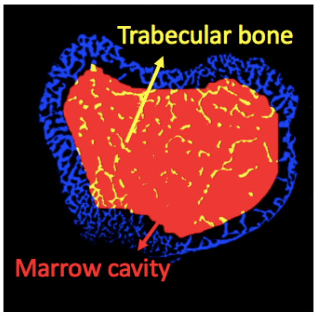

Fig. S5 Illustration of the resulting marrow cavity (red), trabecular bone (yellow), and cortical bone (blue) in one slice
