# Supplementary material for: Impact of Anti-Angiogenic Treatment on Bone Vascularization in a Murine Model of Breast Cancer Bone Metastasis Using Synchrotron Radiation Micro-CT
Source: Cancers (Basel). 2022 Jul 15;14(14):3443. doi: 10.3390/cancers14143443 (PMC9321934; doi:10.3390/cancers14143443)

In this supplementary, we attached all 73 results of trabecular bone in 8 groups (screenshots of 3D volume rendering). Specifically, we considered two time points at 8 days (time point 1) and 22 days (time point 2) after the injection of breast cancer tumor cells. The different treatments of placebo, Bevacizumab, Vatalanib, and combination drugs (Bevacizumab + Vatalanib) were performed at each time point.

Group (T1P): time point 1, placebo

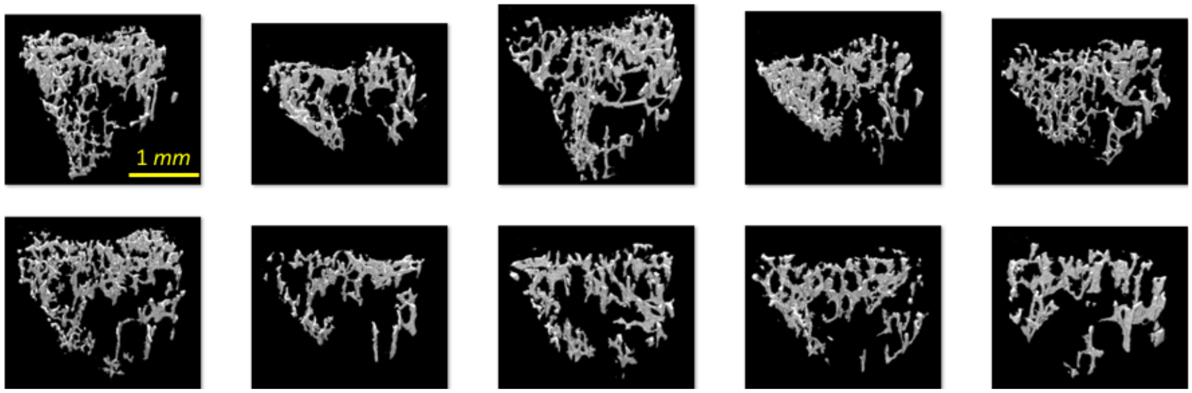

Group (T1B): time point 1, Bevacizumab

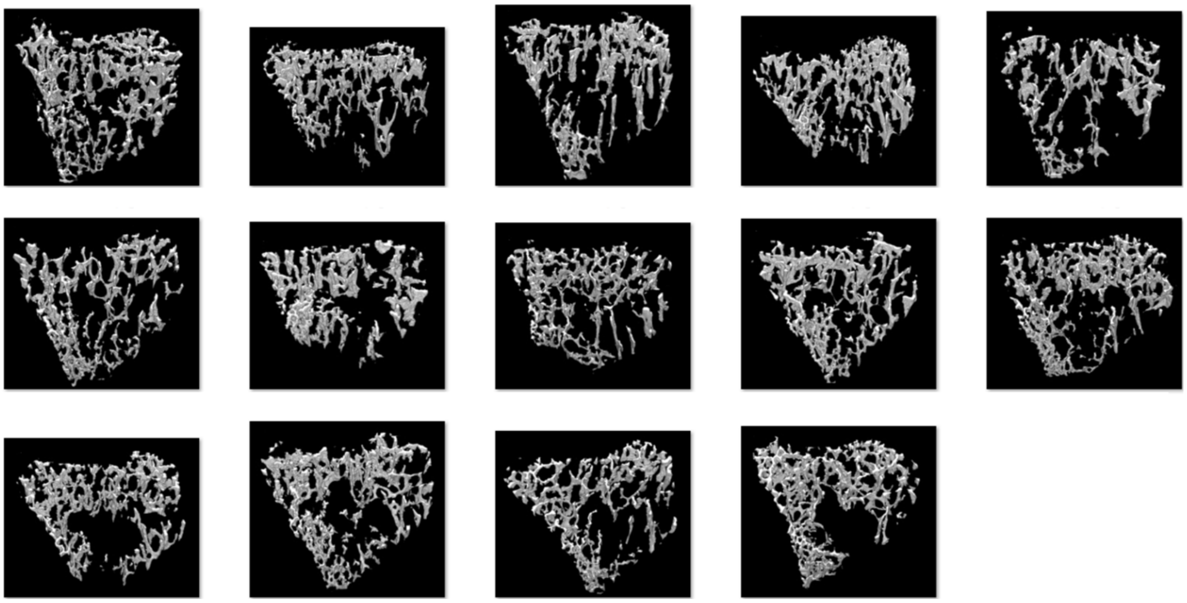

Group (T1V): time point 1, Vatalanib

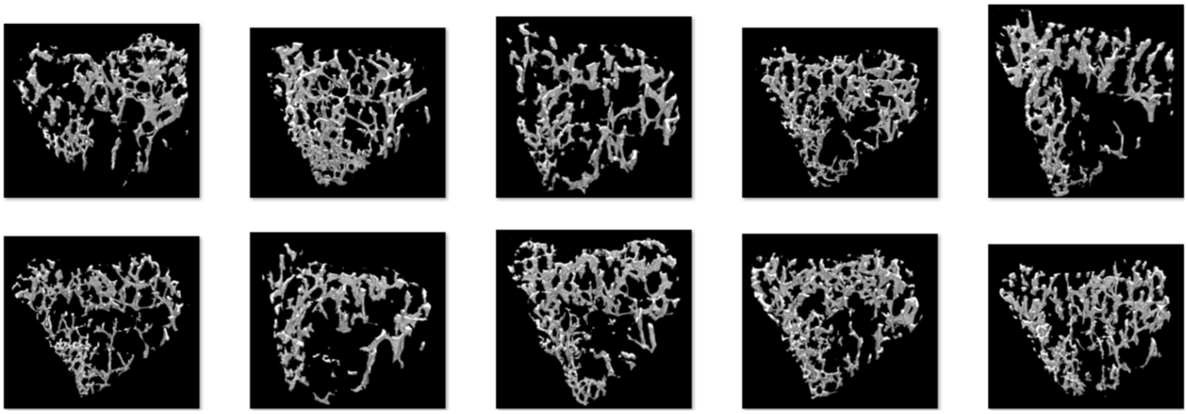

Group (T1C): time point 1, combination (Bevacizumab + Vatalanib)

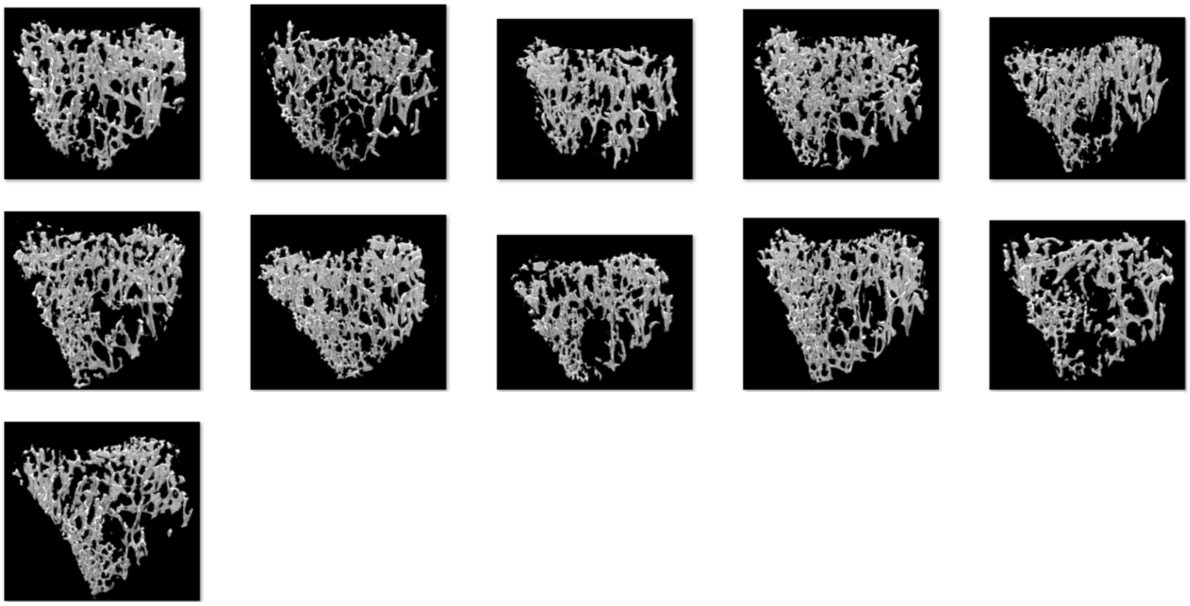

Group (T2P): time point 2, placebo

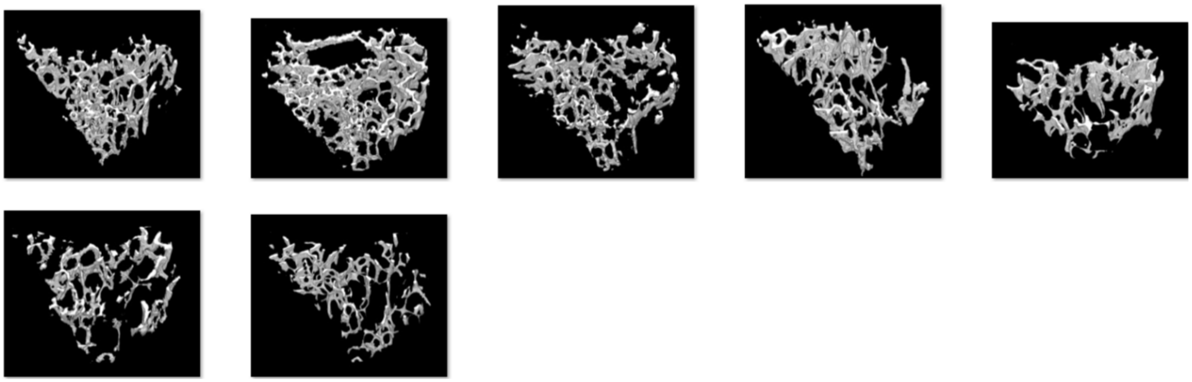

Group (T2B): time point 2, Bevacizumab

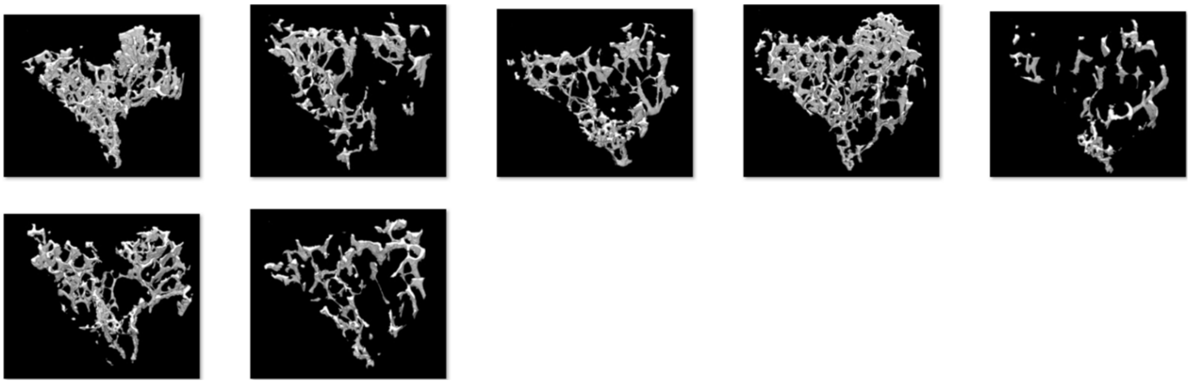

Group (T2V): time point 2, Vatalanib

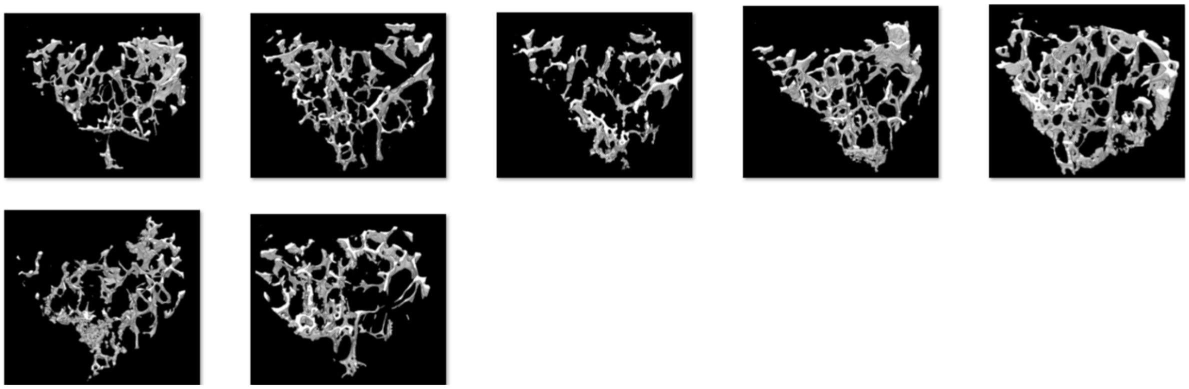

Group (T2C): time point 2, combination (Bevacizumab + Vatalanib)

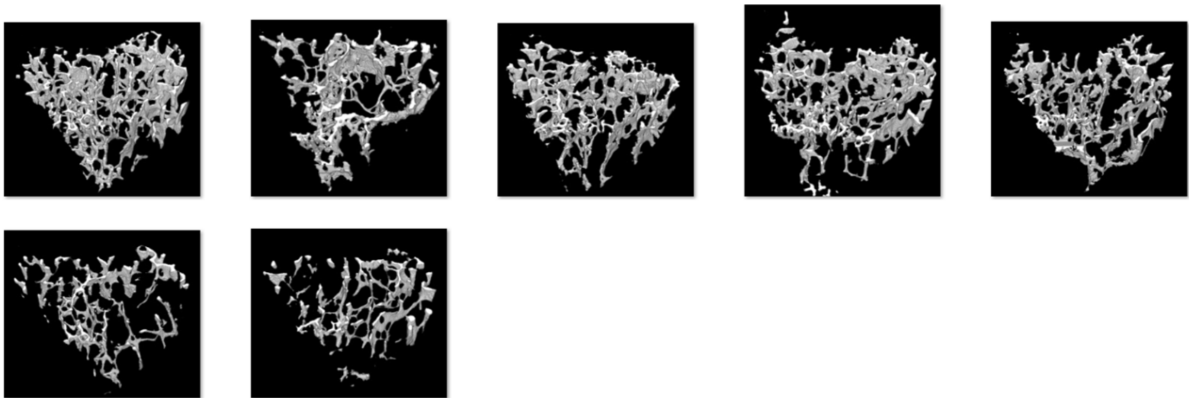

Supplement: Supplementary file 1 [file cancers-14-03443-s001.zip › Supplementary/Supplementary-3.pdf]
