# Supplementary material for: Impact of Anti-Angiogenic Treatment on Bone Vascularization in a Murine Model of Breast Cancer Bone Metastasis Using Synchrotron Radiation Micro-CT
Source: Cancers (Basel). 2022 Jul 15;14(14):3443. doi: 10.3390/cancers14143443 (PMC9321934; doi:10.3390/cancers14143443)

Group (T1P): time point 1, placebo

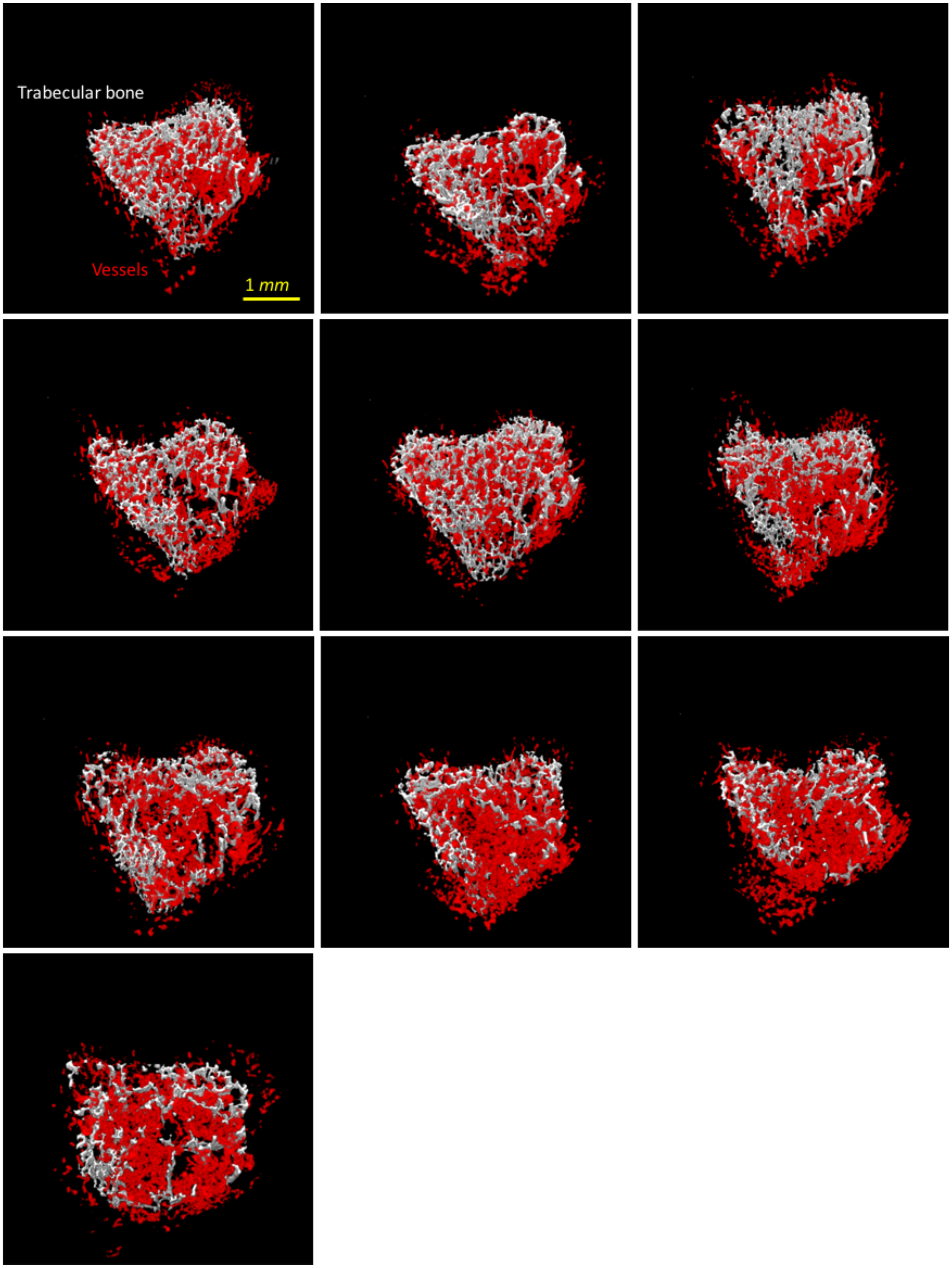

Group (T1B): time point 1, Bevacizumab

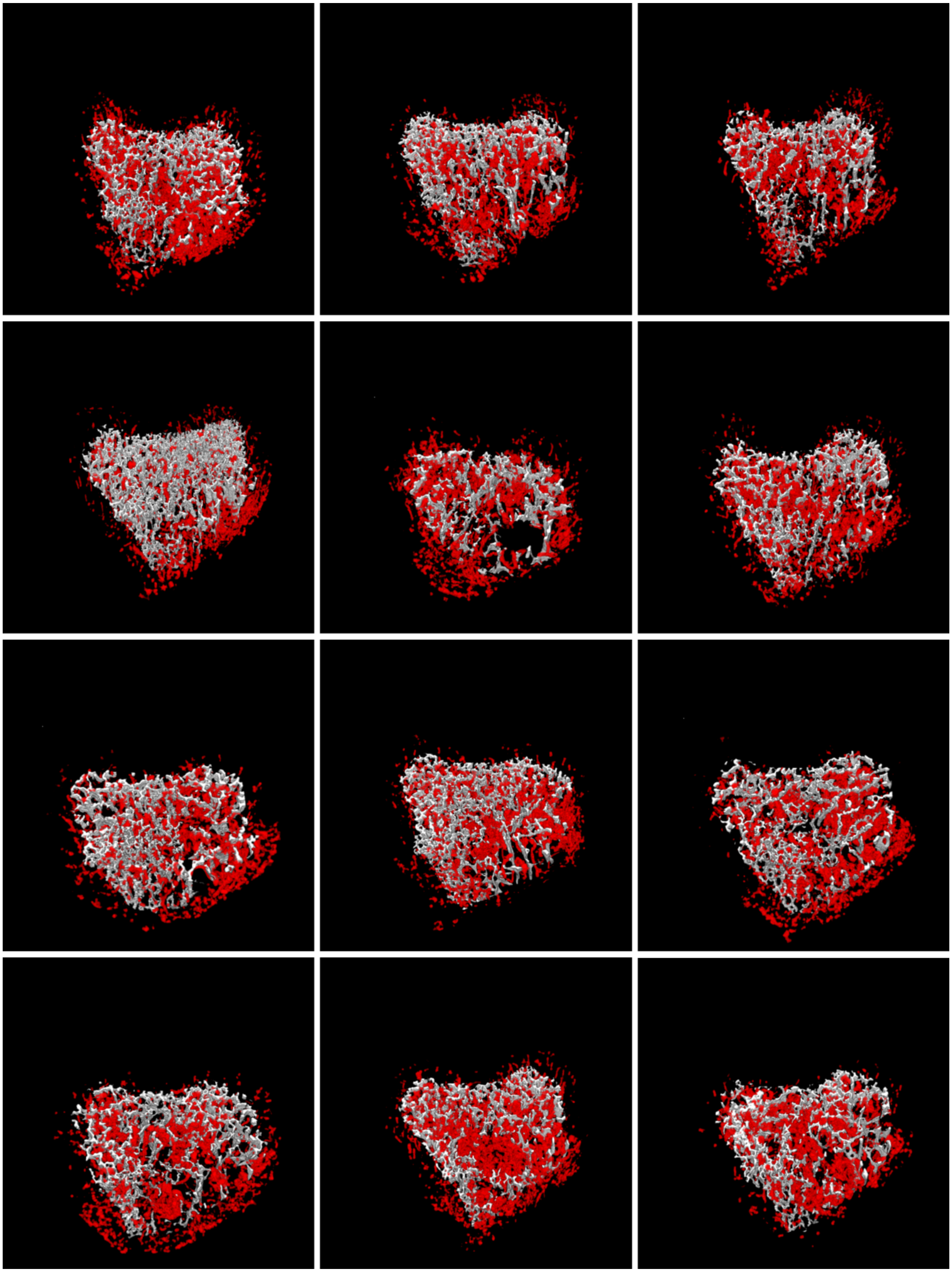

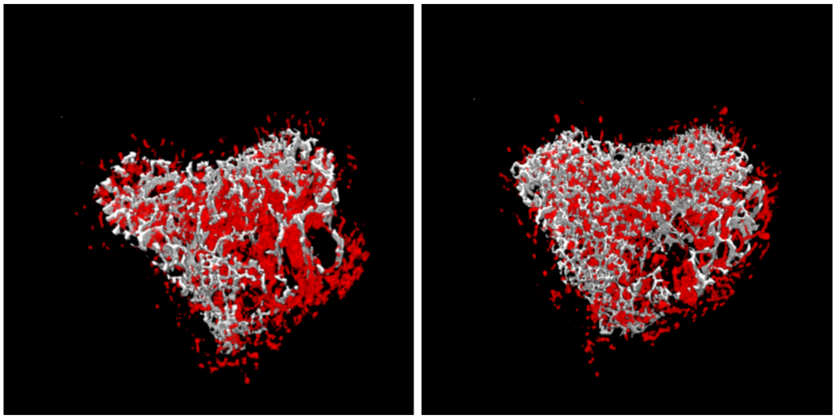

Group (T1V): time point 1, Vatalanib

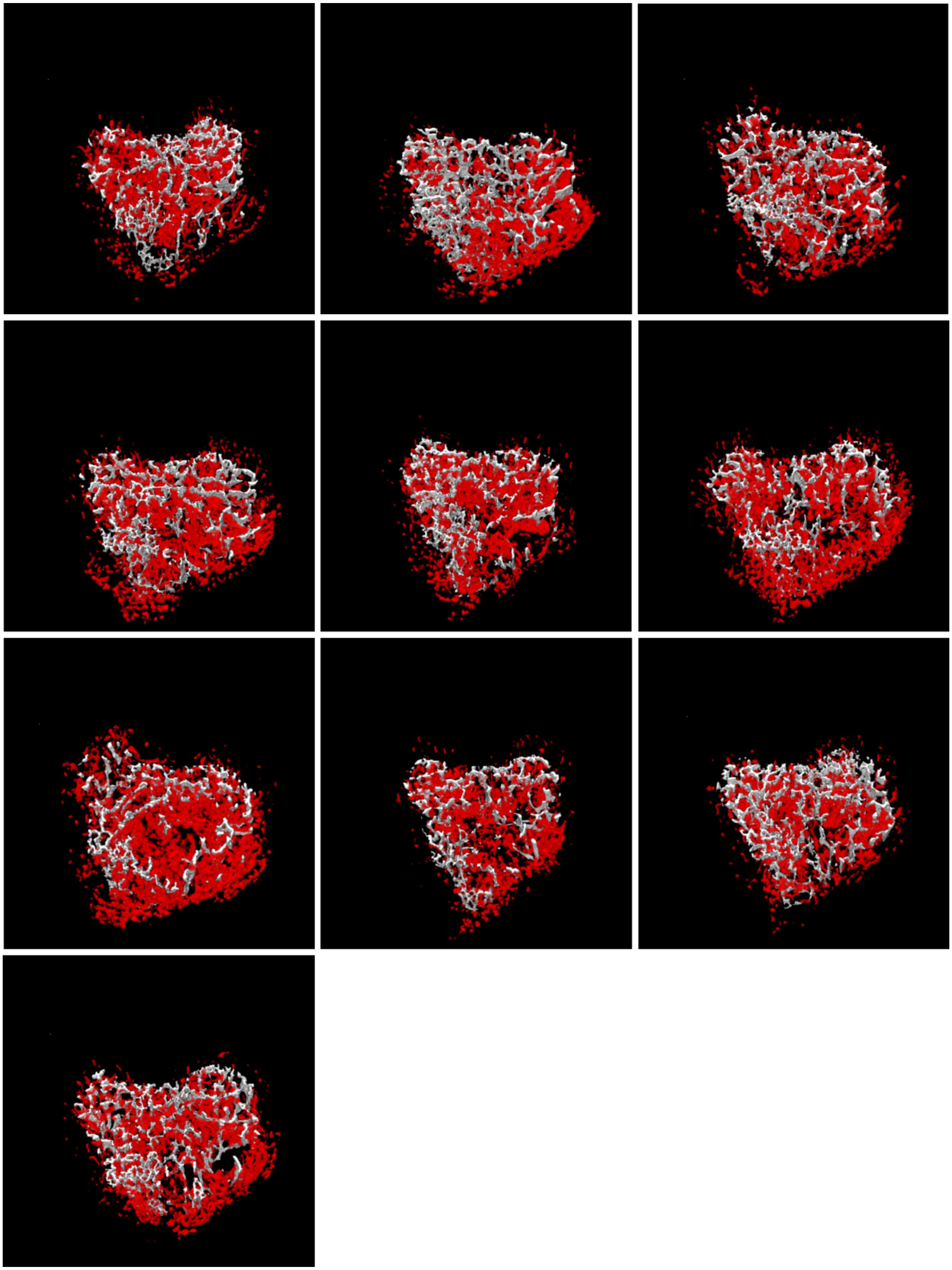

Group (T1C): time point 1, combination (Bevacizumab + Vatalanib)

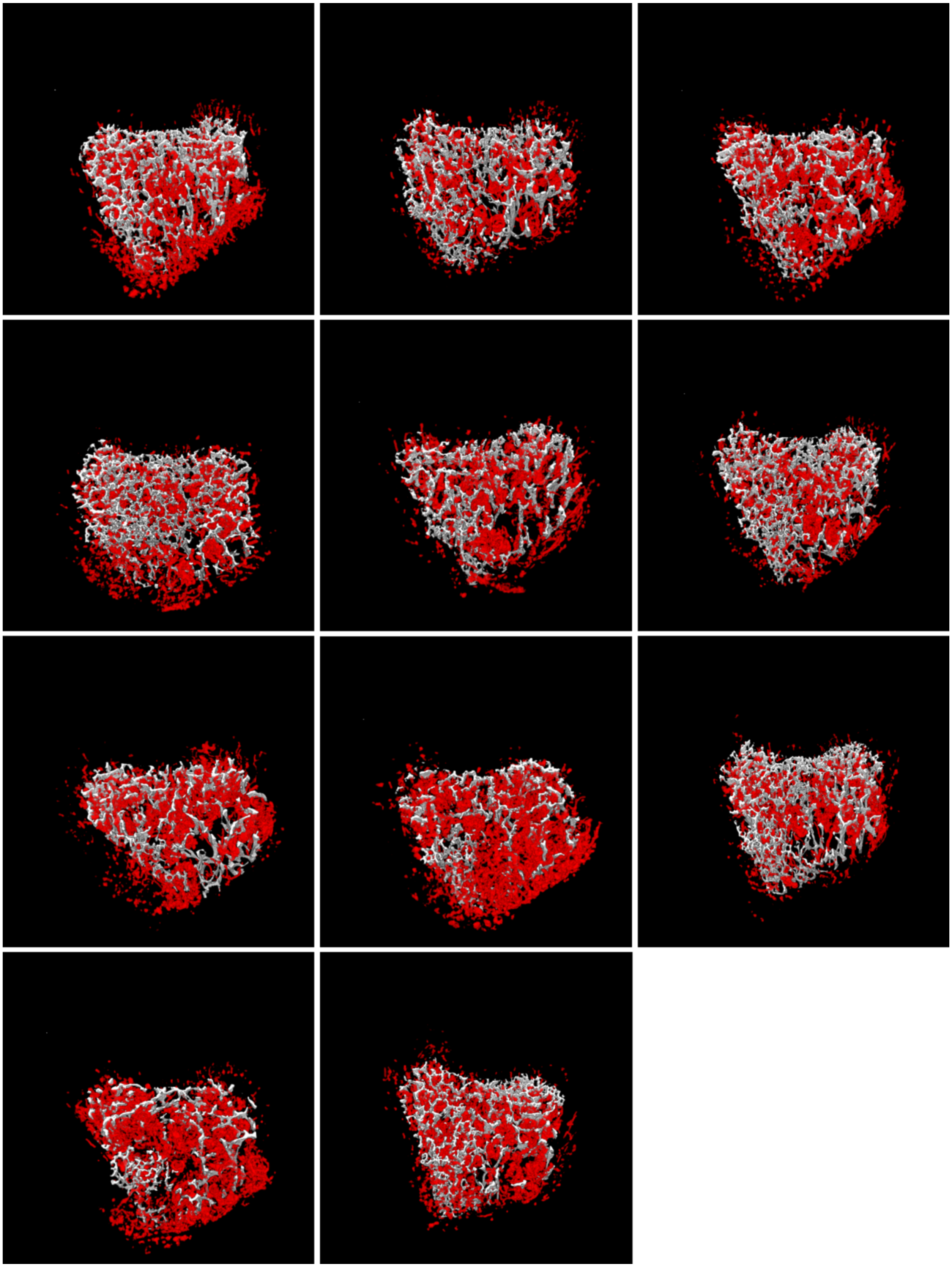

Group (T2P): time point 2, placebo

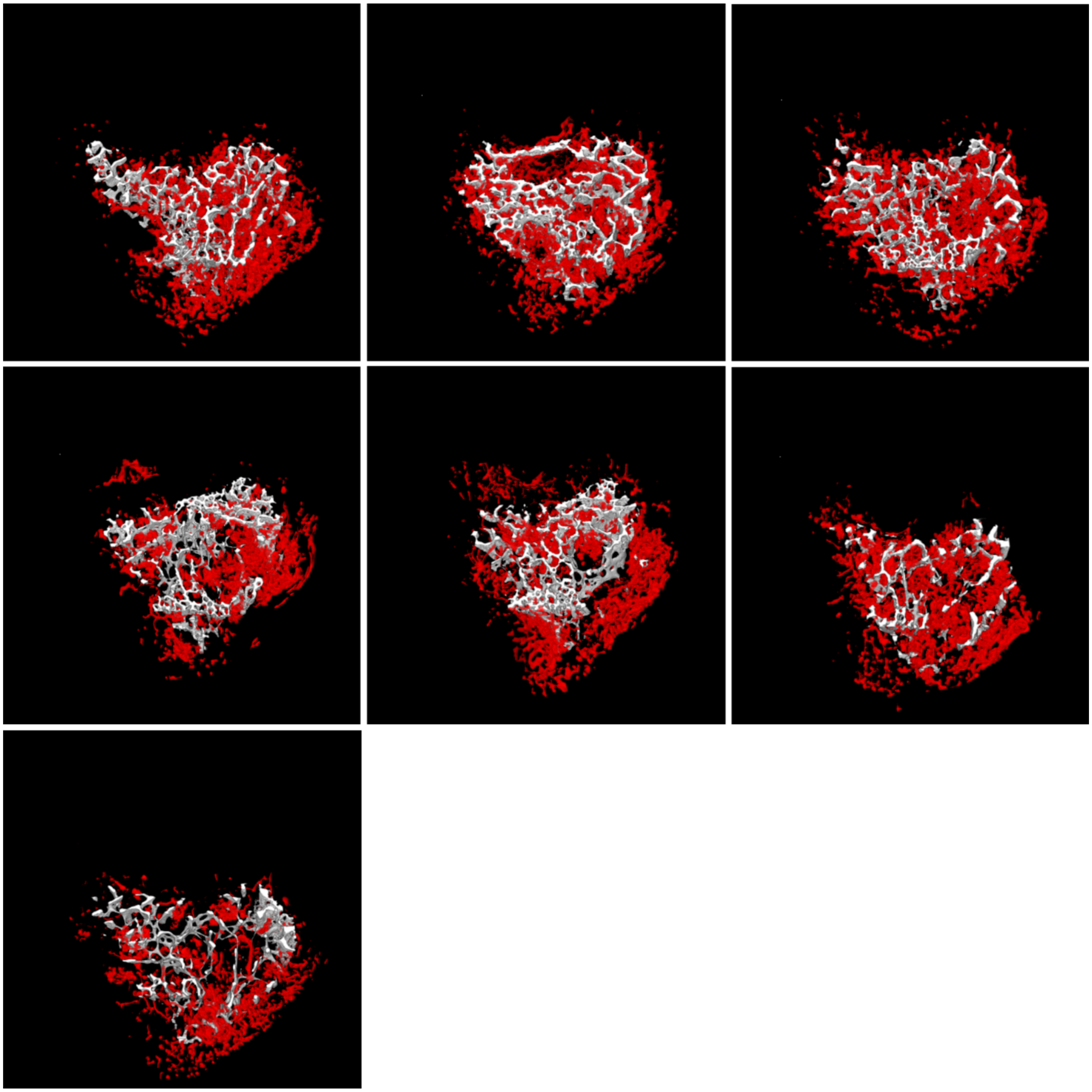

Group (T2B): time point 2, Bevacizumab

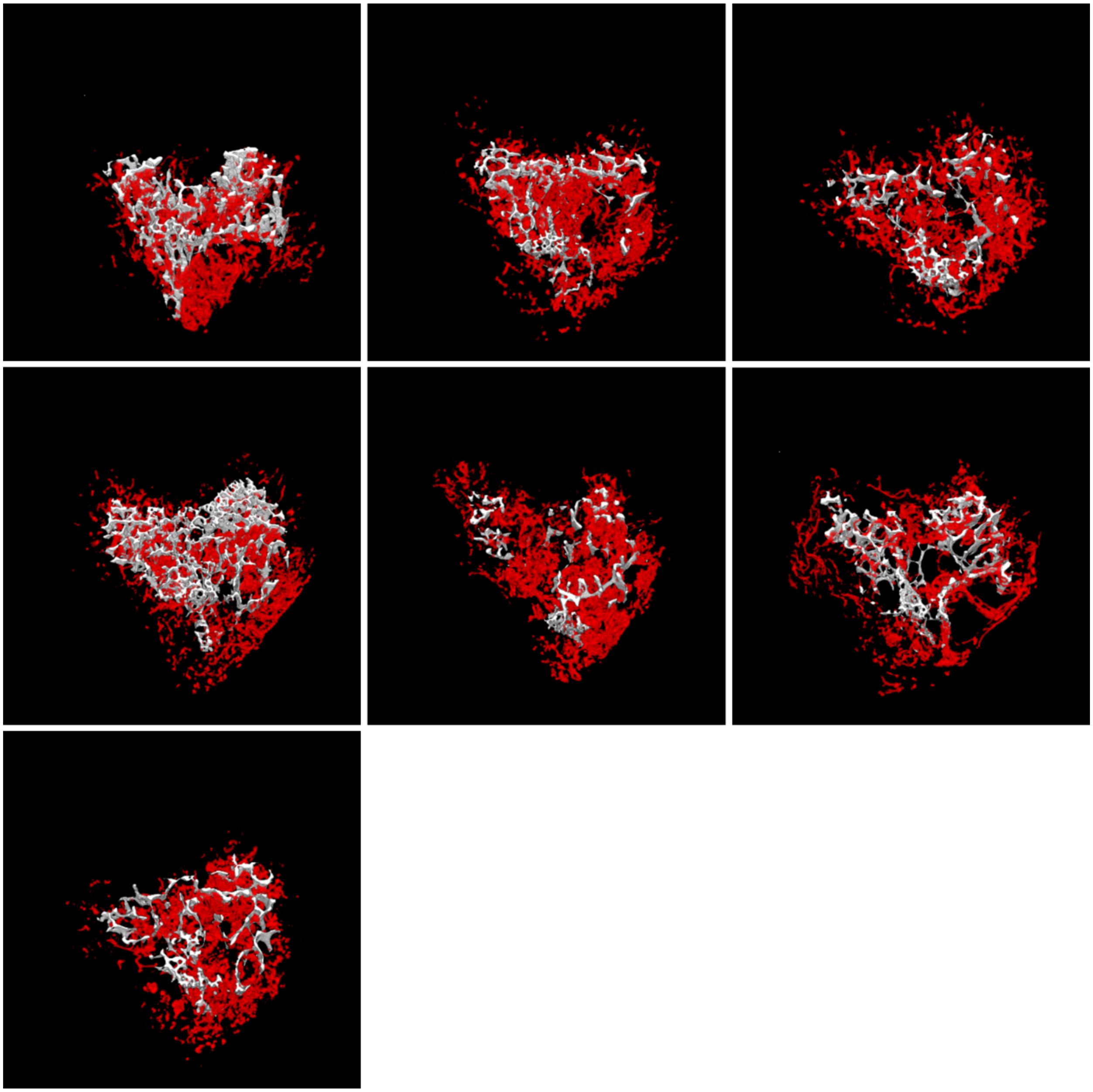

Group (T2V): time point 2, Vatalanib

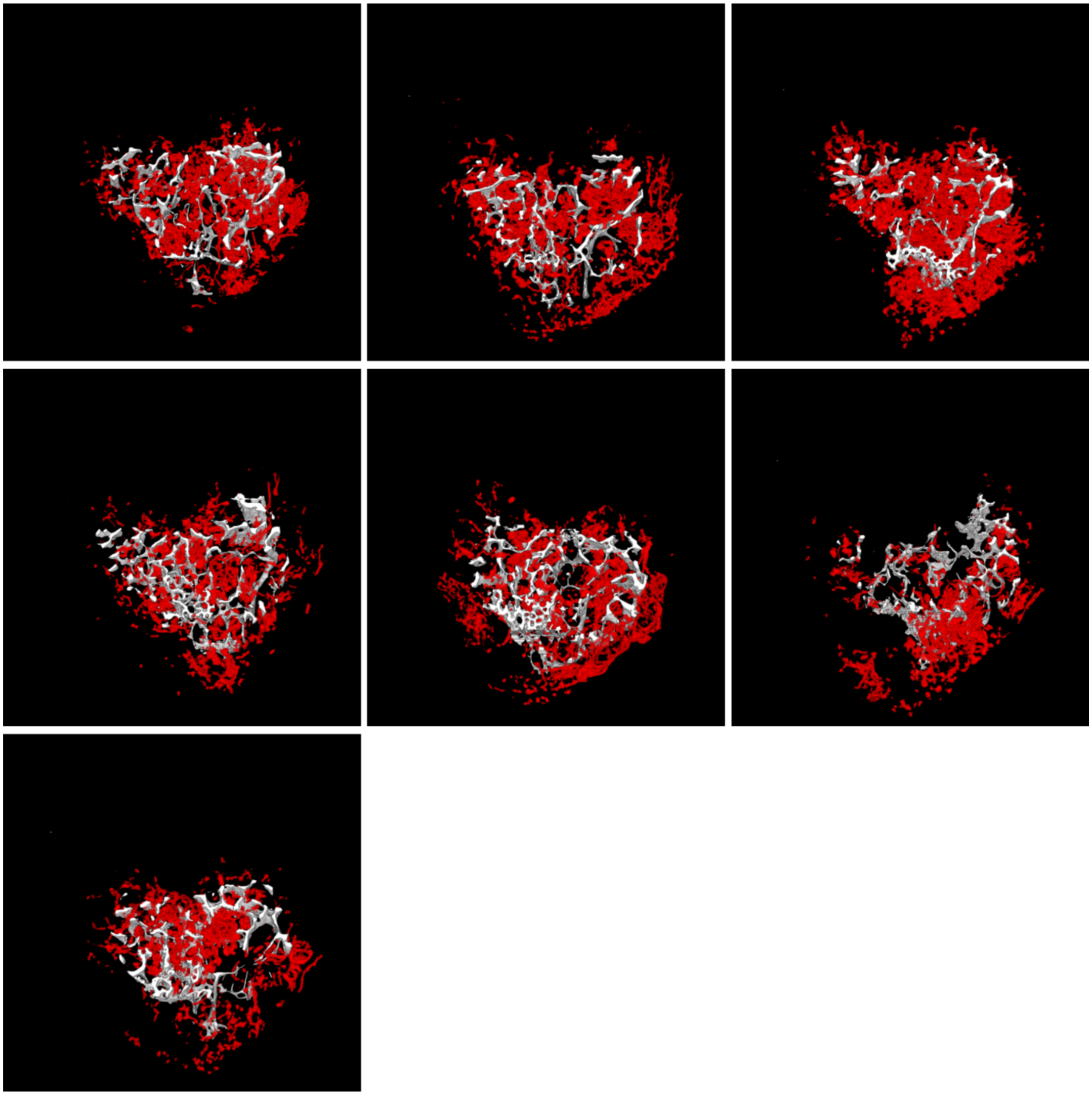

Group (T2C): time point 2, combination (Bevacizumab + Vatalanib)

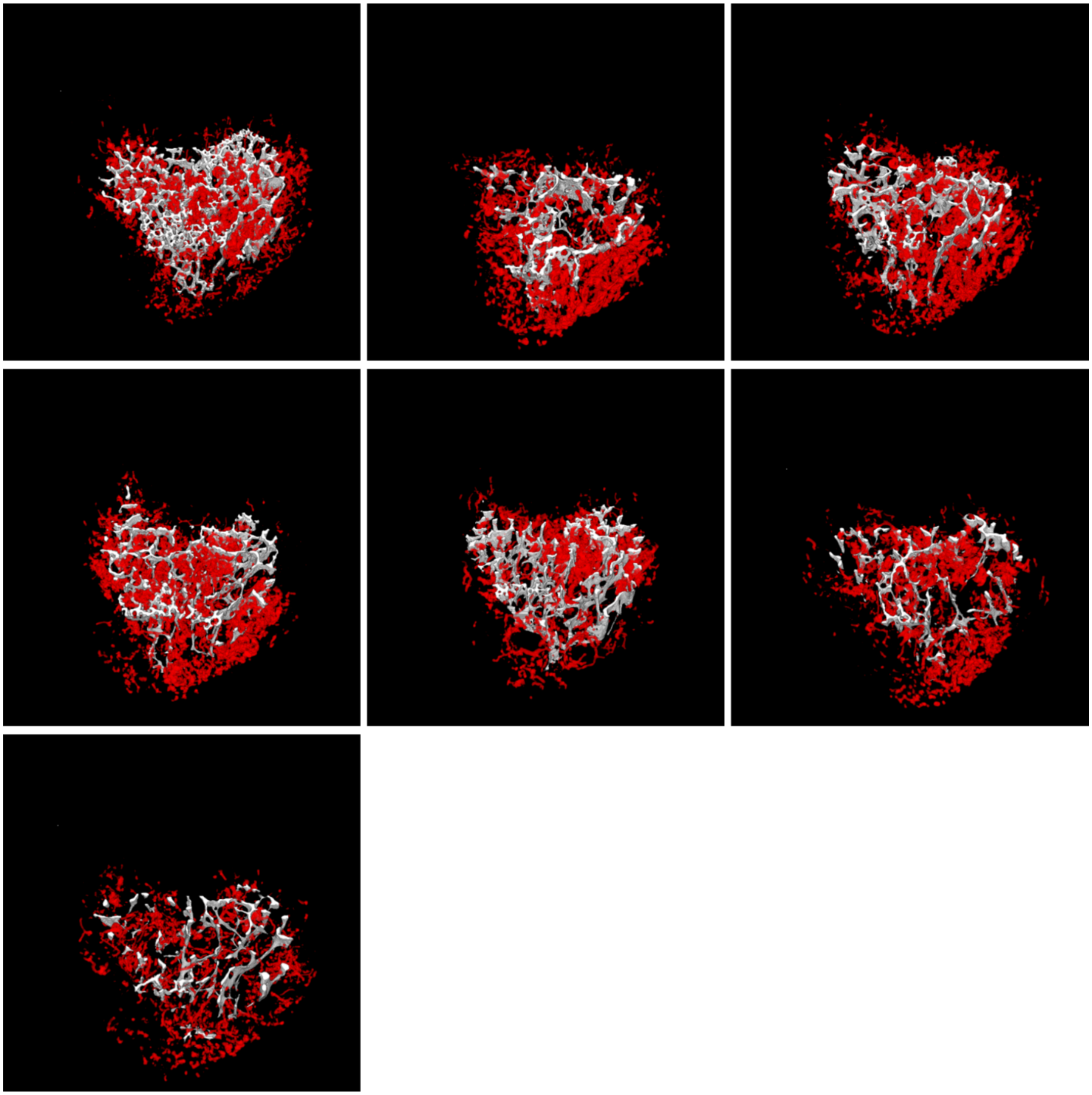

Supplement: Supplementary file 1 [file cancers-14-03443-s001.zip › Supplementary/Supplementary-4.pdf]
